# Okanin Attenuates Mitochondrial Dysfunction and Apoptosis in UVA-Induced HaCaT Cells by Mitophagy Through SIRT3 Pathway

**DOI:** 10.3390/antiox14091040

**Published:** 2025-08-23

**Authors:** Fang Lu, Jiangming Zhong, Qi Zhou, Yiwei Yu, Mengdi Liang, Ying Yuan, Aowei Xie, Jin Cheng, Peng Shu, Jiejie Hao

**Affiliations:** 1 Key Laboratory of Marine Drugs, Ministry of Education, School of Medicine and Pharmacy, Ocean University of China, Qingdao 266003, China; lfangouc@163.com (F.L.); yiweiyu0428@163.com (Y.Y.); liangmengdi0829@163.com (M.L.); sakura03120424@163.com (Y.Y.); xieaowei@stu.ouc.edu.cn (A.X.); amber1285@163.com (J.C.); 2 HBN Research Institute and Biological Laboratory, Shenzhen Hujia Technology Co., Ltd., Shenzhen 518000, China; zhongjiangming@hbn.cn (J.Z.); zhouqi@hbn.cn (Q.Z.); 3 State Key Laboratory Basis of Xinjiang Indigenous Medicinal Plants Resource Utilization, Xinjiang Technical Institute of Physics and Chemistry, Chinese Academy of Sciences, Urumqi 830011, China; 4 University of Chinese Academy of Sciences, Beijing 100049, China

**Keywords:** okanin, mitochondrial dysfunction, sirtuin 3, mitophagy, apoptosis

## Abstract

As the primary bioactive flavonoid in *Coreopsis tinctoria*, okanin has emerged as a promising antioxidant compound of substantial pharmacological interest. However, its efficacy against UVA-mediated photoaging remains unexplored. This research investigated the molecular mechanism underlying the photoprotective activity of okanin against UVA-mediated photoaging. Network pharmacology was employed to predict the pharmacological mechanism of *Coreopsis tinctoria* in skin photoaging, which was then validated through in vivo and in vitro studies. In vitro experiments indicated that treatment with okanin alleviated oxidative damage, apoptosis and mitochondrial dysfunction in HaCaT cells exposed to UVA radiation. In addition, the interaction between okanin and SIRT3 was confirmed using molecular docking, SPR and DARTS assays. However, silencing SIRT3 with siRNA abolished the promoting effects of okanin on mitophagy genes, confirming that okanin protects HaCaT cells against UVA damage through SIRT3 regulation. In in vivo, okanin enhanced the expression of SIRT3 and FOXO3a in dorsal skin, mitigating UV-mediated skin damage. Taken together, our results suggest the protective effects of okanin against UV radiation in both HaCaT cells and mice induced, at least in part, by regulating SIRT3/FOXO3a/PINK1/Parkin signaling pathway. These findings highlight the potential of okanin for use in skin care products aimed at promoting skin repair following UVA exposure.

## 1. Introduction

Ultraviolet (UV) photons primarily target the skin, which serves as the utmost surface separating the human internal body from the external environment, playing a critical role in shielding our organs from damaging solar radiation [1]. Solar UV radiation reaching the Earth’s surface comprises two primary components: medium-wavelength UVB (280–315 nm) and long-wavelength UVA (315–400 nm) [2,3]. In contrast to UVB, UVA can penetrate more deeply into the skin, extending to the stratum basal epidermidis and even affecting the dermal fibroblasts. This deeper penetration induces an instant tanning effect and significantly accelerates skin aging and wrinkling formation [4,5]. Under normal conditions, skin cells maintain a balance of reactive oxygen species (ROS) through their antioxidant systems, protecting against oxidative stress. However, frequent exposure to UV radiation, pollution, and other environmental factors can damage this redox system, leading to oxidative stress [6].

Mitochondria are unique organelles with their own genome, distinct from the nuclear DNA. UV radiation and environment contamination can lead to oxidative stress, leading to the production of excessive ROS, which primarily target mitochondrial DNA (mtDNA). This makes mtDNA especially vulnerable to oxidative damage [6]. In human skin cells irradiated with UVA, oxidative stress markers and DNA damage are significant biological responses. Therefore, maintaining mitochondrial health through quality control mechanisms is crucial for regular cell physiology. It is evident that the quality control processes of intercellular and intracellular regulate the quantity of functional mitochondria. Intracellular quality control encompasses fusion and fission, biogenesis, and selective degradation via mitophagy [7]. Among these, mitophagy is the primary mechanism for eliminating excess or damaged mitochondria [8]. Dysfunctional mitochondria can be harmful to cells [7] as their membranes can rupture, releasing mitochondrial contents like mtDNA and cytochrome c into the cytoplasm, triggering inflammation and apoptosis [9,10]. In response to extrinsic stimulation, like nutritional deficiencies and ROS, mitochondrial membranes can depolarize, leading to damage. Cells selectively package injured mitochondria into autophagosomes for delivery to lysosomes, where they undergo enzymatic degradation [11,12]. This selective degradation process is essential for preserving mitochondrial quality control and energy homeostasis.

Mitochondrial autophagy is coordinately regulated through diverse signaling pathways. Previous studies have identified PINK1/Parkin, BNIP3, NIX and FUNDC1 as key regulatory mechanisms of this process, with PINK1/Parkin being particularly significant [13]. PINK1 and PRKN encode a mitochondrial protein kinase and a ubiquitin ligase, respectively, forming a PINK1/Parkin-dependent regulatory mechanism [14]. PINK1 localizes to the outer membrane of damaged mitochondria, where it recruits and phosphorylates Parkin, an E3 ubiquitin ligase. This process promotes the ubiquitination-mediated degradation and clearance of dysfunctional mitochondria, constituting the canonical Pink1/Parkin pathway [11,15,16].

SIRT3 is a crucial deacetylase that regulates acetylation levels in mitochondria and participates in mitochondrial function, mitophagy, oxidative stress responses, and apoptosis [17,18]. Evidence suggests that SIRT3 is closely linked to mitophagy [19]. It directly interacts with the pivotal transcription factor FOXO3a, activating mitochondrial quality control genes. This interaction enables FOXO3a to adjust PINK1 transcription through conserved binding elements, triggering PINK1/Parkin-mediated mitophagy [20]. Therefore, SIRT3 performs a significant function in the process of PINK1-related mitophagy during cellular stress [21]. Studies indicate that enhancing mitochondrial autophagy can delay UVR-induced skin aging, while increased mitophagy may also inhibit ROS accumulation and cell apoptosis [11].

*Coreopsis tinctoria* Nutt., a North American-native chrysanthemum species with edible properties, is distributed worldwide [22,23]. *Coreopsis tinctoria* Nutt. flowers demonstrate diverse biological activities, exerting neuroprotective, anti-hyperlipidemic, glycemic regulation, and cardioprotective effects [22]. Okanin, a major flavonoid found in *Coreopsis tinctoria* Nutt., has garnered attention for its pharmacological properties [24]. Research by Ganie et al. demonstrated that okanin culminated in a decrease in hyperalgesia and allodynia by regulating the AGEs/NF-κB/Nrf2 pathway [25]. Additionally, Sun et al. found that okanin effectively mitigated UVB-induced epidermal hyperplasia and dermal damage while increasing the collagen content of mice back skin [26]. However, the mechanisms underlying its protective effects against UVA damage require further investigation.

In the current study, network pharmacology and bioinformatic approaches were used to systematically identify and predict the related therapeutic target genes and associated pathways. Our focus is on the effects of okanin on UVA-induced photoaging in both in vivo and in vitro models, specifically examining its antioxidant capacity, impact on mitochondrial dysfunction, mitophagy, and cell apoptosis.

## 2. Materials and Methods

### 2.1. Materials and Reagents

The okanin used in this study was purchased from www.gbw-china.com (Changzhou, China). The Annexin V-FITC Apoptosis Detection kit was purchased from Keygen BioTECH Co., Ltd (Nanjing, China). Mitochondrial respiratory chain complex I (NADH-CoQ) and Π (succinic-CoQ) activity assay kits were bought from Solarbio Science & Technology Co., Ltd. (Beijing, China). GSH assay and T-AOC assay kits were purchased from Nanjing Jiancheng (Nanjing, China). Antibodies against NDUFS1 (#70264), SDHA (#11998), LC3A/B (#12741), SQSTM1/p62 (#39749), PINK1 (#6946), Parkin (#32833), SIRT3 (#2627), FOXO3a (#12829), Phospho-Histone H2A.X (#9718), Bax (#5023), Bcl-2 (#3498), Cytochrome c (#11940) and caspase-3 (#14220) were purchased from CST (Beverly, MA, USA).

### 2.2. Cell Culture and UVA Irradiation

HaCaT cells (the Chinese Academy of Sciences Cell Bank, Shanghai, China) were maintained in DMEM supplemented with 10% FBS and 1% penicillin/streptomycin at 37 °C/5% CO_2_. Cells between passages 3 and 7 were used for all experiments. HaCaT cells were plated in 96- or 6-well plates for 24 h at 37 °C. Subsequently, the cells were treated with okanin (5, 10, and 20 μM) in phenol red-free DMEM, and the control and UVA group were replaced with phenol red-free DMEM containing the same concentration of DMSO and then exposed to UVA radiation after removing plate lid. Next, the cells were incubated for 24 h post UVA radiation. Then, HaCaT cell viability was evaluated using the MTT assay.

### 2.3. Measurement of LDH, GSH, T-AOC and ROS

Cell supernatants and lysates were detected for LDH release, intracellular GSH, and T-AOC according the manufacturer’s protocols.

The intracellular ROS levels after UVA exposure were measured using DCFH-DA. HaCaT cells pretreated with okanin were irradiated and incubated with DCFH-DA. Finally, fluorescence was quantified (Ex/Em: 488/535) through a microplate reader (Molecular Devices Co., Ltd, Shanghai, China.

### 2.4. Measurement of Mitochondrial Membrane Potential (MMP)

MMP was assessed using JC-1 probe. HaCaT cells were incubated with JC-1 at 37 °C for 30 min and analyzed by a microplate reader (monomers: Ex/Em 490/530; aggregates: Ex/Em 525/590). ΔΨm was determined as the aggregate/monomer ratio.

### 2.5. TEM Analysis of Mitochondria

HaCaT cells cultured in the 6-well plate were scraped and centrifuged at 1200 rpm for 5 min. After discarding supernatant, glutaraldehyde was added and fixed at 4 °C overnight. And then mitochondrial morphological changes were analyzed by TEM (JEM-100CX II) (JEOL, Beijing, China).

### 2.6. Flow Cytometry

We used the Annexin V-FITC/PI assay kit to assess cell apoptosis. Cells were harvested, stained with Annexin V-FITC and PI for 30 min in the darkness, and analyzed on a BD flow cytometer (BD Biosciences, Franklin Lakes, NJ, USA). Data were processed with Flow Jo software (10.8.1).

### 2.7. siRNA Studies

HaCaT cells at about 50% confluence were transfected with 50 nM siRNA using RNAFit (HANBI Biotechnology, Shanghai, China) based on the manufacturer’s guideline protocol. After 48 h, followed by treatment, cells were treated with or without 5 μM okanin for 24 h and then harvested for further research. The *si-SIRT3* sequences are listed in Table 1.

### 2.8. Western Blot Analysis

The RIPA buffer containing inhibitors of phosphatases and proteases was used to homogenize the protein lysates of the cells. Proteins (20 μg proteins per lane) were resolved on 10% SDS-PAGE and electro-transferred to the nitrocellulose membrane. Following 2 h blocking (5% BSA/TBST, RT), membranes were incubated overnight at 4 °C with antibodies. Following 5 TBST washes, membranes were probed with an AP-marked secondary antibody (1 h, RT). BCIP/NBT colorimetric detection was performed. Data represent the percentage of control values, analyzed using Image J (1.8.0).

### 2.9. Quantitative PCR

RNA extraction was performed with SparkZol reagent (Spark Jade Co., Ltd, Jinan, China), followed by cDNA synthesis from 2 µg RNA using Hiscript III RT Super Mix (Vazyme Co., Ltd, Nanjing, China). Next, gene expression analysis was conducted via real-time PCR (Quant Studio TM 3, Thermo Fisher Scientific, MA, USA) with the following thermal profile: initial denaturation at 95 °C for 3 min and then 40 cycles of 95 °C for 10 s and 60 °C for 30 s. Data were normalized to β-actin and analyzed using 2^−ΔΔCT^ research, with primer sequences provided in Table 2.

### 2.10. Formulation of Okanin Cream

As shown in Table 3, oil-phase components (stearic acid, white petrolatum, and liquid paraffin) were combined in a beaker and heated to 100 °C to achieve complete melting. Concurrently, aqueous-phase components (Tween-80, glycerol and distilled water) were heated to 100 °C in a separate beaker. Under continuous stirring, we added the heated aqueous phase to the molten oil phase. Subsequently, okanin was added to the emulsion, which was maintained at a constant temperature using a water bath and stirred to ensure homogeneity. Finally, the mixture was stirred at room temperature until condensation occurred.

### 2.11. Animal Models

We purchased 6–8-week-old female BALB/c mice from Pengyue Laboratory Animal Co., Ltd. (Jinan, China), and cultivated them under controlled conditions (25 °C, 12 h light/dark cycle) with free access to water and food. Before the experiment, mice had their dorsal hair shaved and underwent 3 days of environmental acclimatization. BALB/c mice were allocated into 5 groups at random (n = 8 per group): (1) control, (2) UV radiation + vehicle, (3) UV radiation + 20 mg/kg/d lipoic acid, (4) UV radiation + 0.5 mg/kg/d okanin (low dose), and (5) UV radiation + 1.5 mg/kg/d okanin (high dose).

We utilized 3 UVA (340 nm peak) and 2 UVB (313 nm peak) lamps with alternating arrangements to imitate UV irradiation. An ultraviolet meter (TS280E) (Shenzhen Yuchen Instrument Equipment Co., Ltd, Shenzhen, China) was used to assess UV irradiance. The lamps were positioned about 40 cm above the mice. The mice received daily irradiation (1 h/day) every other day for a total of 15 days, achieving cumulable doses of 10 J/cm^2^ (UVA) and 0.63 J/cm^2^ (UVB). Mice received a dorsal application of okanin formulation preceding irradiation. All procedures adhered to ethical guidelines for animal use reduction and discomfort minimization.

### 2.12. Statistical Analysis

All data are based on at least three independent experiments (n ≥ 3). Data are expressed as mean ± SEM. A one-way ANOVA was conducted using GraphPad Prism 8.0.2 (GraphPad Software). *p*-values < 0.05 denote statistical significance.

## 3. Results

### 3.1. Identification of Potential Targets for Coreopsis tinctoria Nutt. in Treating Skin Photoaging

**Figure 1:**
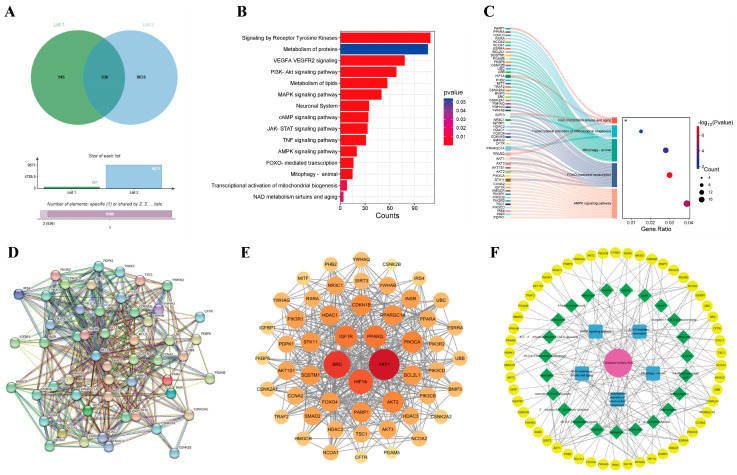
Identification of therapeutic targets for *Coreopsis tinctoria* Nutt. against skin photoaging. (**A**) Venn diagram of action targets of *Coreopsis tinctoria* Nutt. and related targets of skin photoaging. List 1 represents targets related to active ingredients of *Coreopsis tinctoria* Nutt., List 2 represents the targets of skin photoaging, and overlapping part represents intersection targets. (**B**) An enrichment classification diagram of core targets. (**C**) Sankey and dot plot of the core targets and pathways. (**D**) PPI network of potential targets of *Coreopsis tinctoria* Nutt. in treating skin photoaging. (**E**) Key genes for *Coreopsis tinctoria* Nutt. treatment of skin photoaging. (**F**) *Coreopsis tinctoria* Nutt. drug-active ingredients-targets-pathway network.

We searched drug-related databases using *Coreopsis tinctoria* Nutt. as a keyword to identify the targets, yielding 681 targets after duplicate removal. Skin photoaging-related targets were then retrieved from the GeneCards database, with a screening threshold set at a relevance score ≥ medium. Subsequently, 9571 targets were obtained through conditional screening. A Venn diagram analysis identified 536 overlapping genes between *Coreopsis tinctoria* Nutt. targets and photoaging-related genes, suggesting potential therapeutic targets for *Coreopsis tinctoria* Nutt. in photoaging treatment (Figure 1A). The methods of network pharmacology can be found in the Supplementary Materials.

### 3.2. Identification of Key Signaling Pathways in Intersection Targets

To understand the signaling pathways of the intersection targets, we performed WIKIPATHWAYS, REACTOME and KEGG functional analyses, identifying 1132 enriched pathways (Figure 1B). In addition to pathways such as Receptor Tyrosine Kinases, the metabolism of proteins, VEGFA VEGFR2 signaling and the PI3K-Akt signaling pathway, etc., we also found pathways related to mitochondrial function and autophagy, including the AMPK signaling pathway, FOXO-mediated transcription, mitophagy in animals, the transcriptional activation of mitochondrial biogenesis, and NAD metabolism, sirtuins, and aging. We selected the mitochondrial function and autophagy pathway as the core pathway and compiled the genes on the mitochondrial pathway as primary targets for further study (Figure 1C).

### 3.3. Analysis of Protein–Protein Interaction (PPI) Network for Coreopsis tinctoria Nutt. in Photoaging Treatment

The STRING database was utilized to construct the PPI network to visually analyze potential targets of *Coreopsis tinctoria* Nutt. in treating photoaging (Figure 1D), demonstrating significant connectivity and confidence levels with a p-value enrichment of <1.0 × 10^−16^. Subsequently, topological analysis using Cytoscape 3.10.3 identified numerous targets of the network’s node degree (Figure 1E), including *AKT1, SRC, SQSTM1, PPARGC1A, BCL2L1 and SIRT3*, which exhibit higher degrees, underscoring their significance in the potential therapeutic effects of *Coreopsis tinctoria* Nutt. on photoaging.

### 3.4. Drug-Active Ingredient–Target–Pathway Network Construction

We set the size of nodes in the network diagram according to their scores from small to large. A pharmacological network was constructed to map the relationship between *Coreopsis tinctoria* Nutt. and its active ingredients, targets, and pathway. As shown in Figure 1F, the results suggested that *Coreopsis tinctoria* Nutt. might exert anti-photoaging effects through signaling pathways such as mitophagy in animals, the transcriptional activation of mitochondrial biogenesis, NAD metabolism, sirtuins, and aging, the AMPK signaling pathway, and FOXO-mediated transcription.

### 3.5. Okanin Enhances HaCaT Cell Viability Exposed to UVA

**Figure 2:**
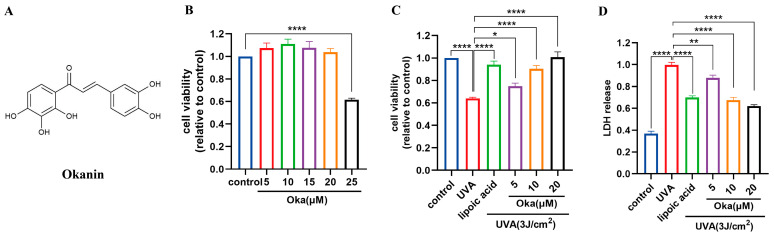
Okanin protects against the UVA-induced decrease in HaCaT cell viability. (**A**) The chemical structure of okanin. (**B**) HaCaT cell viability was evaluated using the MTT assay after incubation with different concentrations of okanin (n = 6). (**C**) HaCaT cells were treated with different concentrations of okanin and exposed to UVA (3 J/cm^2^) (n = 6). (**D**) LDH release (n = 6). Values are presented as the mean ± SEM. * *p* < 0.05, ** *p* < 0.01, **** *p* < 0.0001.

Based on previous work in our laboratory, we chose a UVA of 3 J/cm^2^ for further studies. Cytotoxicity testing using the MTT assay revealed that okanin (Figure 2A) at concentrations of 20 μM had no toxic effect on HaCaT cells (Figure 2B). Lipoic acid, a well-known mitochondrial antioxidant, served as the positive control (100 μM) in this study. Treatment with okanin remarkably enhanced UVA-exposed HaCaT cell viability (Figure 2C). Furthermore, UVA exposure increased LDH release, which was dose-dependently attenuated by okanin treatment (Figure 2D).

### 3.6. Okanin Reduces ROS Production and Enhances Antioxidant Effect in HaCaT Cells Exposed to UVA

**Figure 3:**
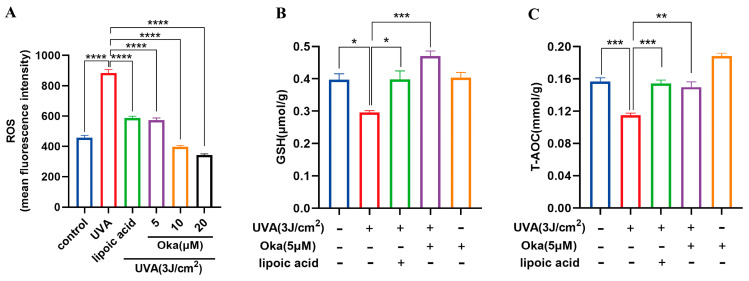
Okanin enhances the antioxidant capacity of UVA-induced HaCaT cells. (**A**) The cells were incubated with 10 μM DCFH-DA and incubated for the ROS production assay (n = 6). (**B**) GSH and (**C**) T-AOC levels (n = 6). Values are presented as the mean ± SEM. * *p* < 0.05, ** *p* < 0.01, *** *p* < 0.001, **** *p* < 0.0001.

UVA radiation significantly augmented ROS production in HaCaT cells, which was effectively attenuated through okanin treatment (Figure 3A).

Relative to the controls, UVA exposure significantly decreased the GSH generation, while okanin treatment reversed the depletion of intracellular GSH (Figure 3B). These findings suggest that okanin mitigates UVA-induced ROS overproduction and improved T-AOC radiated (Figure 3C) might through improving GSH levels.

### 3.7. Okanin Enhanced Mitochondrial Function in HaCaT Cells Exposed to UVA

**Figure 4:**
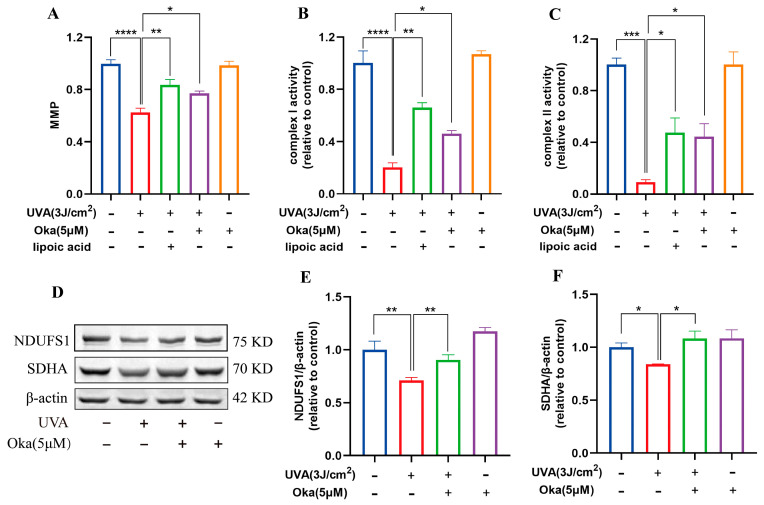
Okanin improves the mitochondrial function of UVA-induced HaCaT cells. (**A**) The HaCaT cells were incubated with JC-1 for the mitochondrial membrane potential (n = 5). (**B**,**C**) Activities of complexes Ⅰ and Ⅱ (n = 5). (**D**) Representative bands of NDUFS1 and SDHA proteins. The corresponding quantification of NDUFS1 (**E**) and SDHA (**F**). Values are presented as the mean ± SEM. * *p* < 0.05, ** *p* < 0.01, *** *p* < 0.001, **** *p* < 0.0001.

As displayed in Figure 4A, okanin treatment significantly attenuated UVA-mediated MMP loss in HaCaT cells. It has been established that mitochondrial respiratory chain complexes I and II are important for mitochondrial function. We then assessed the effects of okanin on mitochondrial function radiated by UVA. In UVA-radiated HaCaT cells, okanin treatment restored the activity of mitochondrial respiratory chain complex I and II activities (Figure 4B,C). The Western blotting analysis revealed the UVA-induced downregulation of mitochondrial respiratory chain complex-associated proteins NDUFS1 and SDHA, which okanin reversed (Figure 4D–F). These results demonstrated that okanin could enhance mitochondrial function.

### 3.8. Okanin Improved Mitochondrial Morphology in HaCaT Cells Exposed to UVA

**Figure 5:**
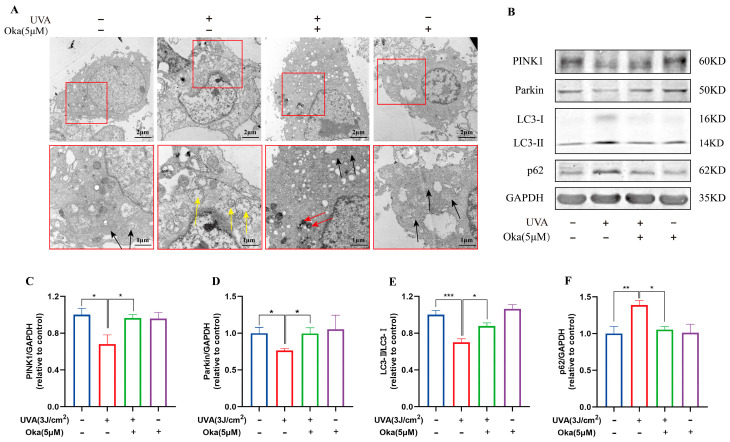
Okanin improves the mitochondrial function of UVA-induced HaCaT cells. (**A**) Mitochondria ultrastructure changes after UVA irradiation by TEM scanning (scale bars: 2 μm and 1 μm). The yellow arrow indicates severe mitochondrial vacuolation within cells. The black arrow indicates normal mitochondria. The red arrow indicates the autophagosome engulfment of damaged mitochondria. (**B**) Representative bands of PINK1, Parkin LC3 and p62 proteins. The corresponding quantification of PINK1 (**C**), Parkin (**D**), LC3 (**E**) and p62 (**F**). Values are presented as the mean ± SEM. * *p* < 0.05, ** *p* < 0.01, *** *p* < 0.001.

The modulation of mitochondrial morphology may be an important feature of its protective function. We observed alterations in mitochondrial morphology using TEM following different treatments. As displayed in Figure 5A, UVA-exposed cells exhibited a destroyed mitochondrial structure, abnormal shapes, and damaged cristae. Notably, okanin treatment preserved mitochondrial integrity and markedly restored morphology, indicating its dual protective effects on mitochondrial morphology and function.

A further analysis of mitophagy-associated proteins revealed UVA-induced dysregulation in HaCaT cells, such as reduced LC3-II/I, PINK1 and Parkin with concomitant p62 accumulation (Figure 5B), which indicated impaired mitophagy. Okanin effectively restored these effects by promoting LC3-II conversion, PINK1/Parkin expression, and p62 degradation (Figure 5C–F). Taken together, the above evidence suggested that okanin exerted mitophagy induction in HaCaT cells with UVA injury through the upregulation of the PINK1/Parkin pathway.

### 3.9. Molecular Docking and Molecular Dynamics Between Okanin and SIRT3

**Figure 6:**
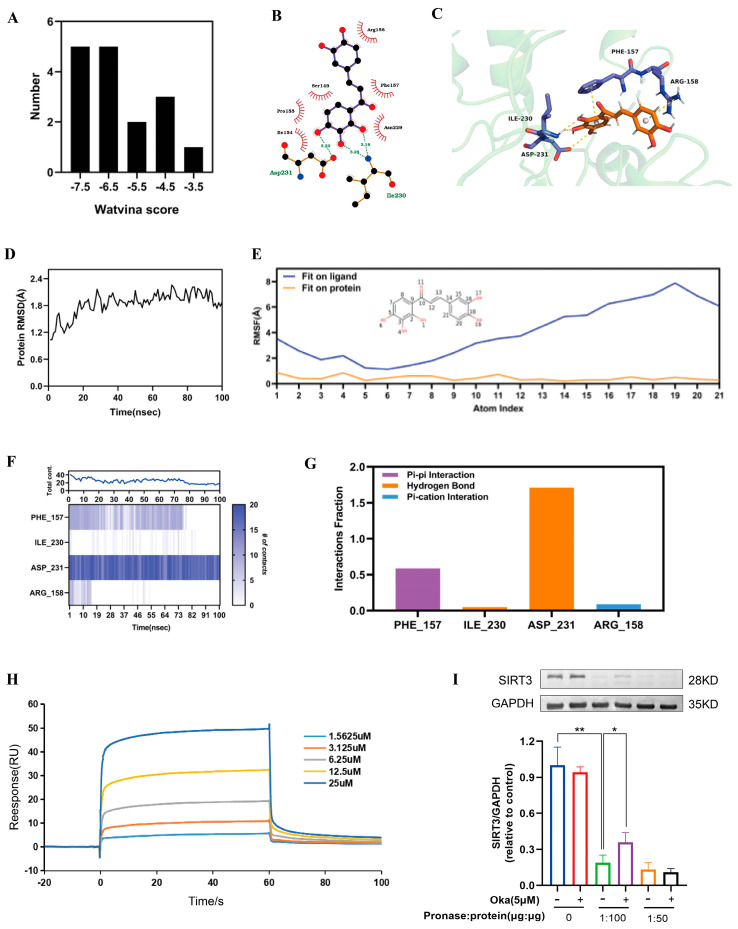
The interaction of okanin with SIRT3. (**A**) The docking score distribution diagram of okanin and 17 crystal structures of SIRT3. (**B**) The 2D interaction structures between okanin and SIRT3. Hydrogen bonds are shown as green dotted lines, while the spoked arcs represent residues making nonbonded contacts with the ligand. (**C**) The 3D interaction structures between okanin and SIRT3. (**D**) The Root Mean Square Deviation (RMSD) of the SIRT3 backbone. (**E**) The Ligand Root Mean Square Fluctuation (L-RMSF) of okanin in SIRT3. (**F**) The interactions between key residues and SIRT3 during the whole simulation process. (**G**) The main interaction fractions between okanin and SIRT3. (**H**) The binding affinity between okanin and SIRT3, with KD = 4.977 × 10^−6^. (**I**) The direct interaction between SIRT3 and okanin was studied by using a DARTS assay. Values are presented as the mean ± SEM. * *p* < 0.05, ** *p* < 0.01.

As shown in Supplementary Materials, we used molecular docking to analyze okanin and SIRT3 interactions. We acquired 17 SIRT3 crystal structures from the PDB database. Figure 6A displays the docking score distribution between the 17 SIRT3 crystal structures and okanin. The biggest affinity interaction occurred with a score of −7.94 between SIRT3 and okanin (Figure 6B,C). To further evaluate the binding stability of okanin with SIRT3 (PDB ID: 4JT8), molecular dynamics (MD) simulations were performed. The SIRT3 backbone maintained structural integrity (RMSD < 2.4 Å) throughout the simulation (Figure 6D). Okanin exhibited a stable combination with the protein pocket, with heavy atom RMSF values < 2 Å relative to SIRT3 (Figure 6E). PHE157, ILE230 and ASP231 were critical binding residues. PHE 157 and ASP231 remained consistent with okanin, evidenced by stable pi–pi (0.6) and hydrogen bond (1.7) fractions (Figure 6F,G).

### 3.10. Binding Affinity of Okanin to SIRT3

Next, SPR analysis revealed dose-dependent binding between okanin and SIRT3 (Figure 6H), with a calculated KD of 4.977 μM, thereby proving their interaction.

The DARTS assay exploits the reduced protease susceptibility of drug-bound target proteins upon drug binding, providing an important approach for detecting drug–protein interactions [27]. The DARTS experiments demonstrated that okanin treatment significantly enhanced SIRT3 resistance to pronase digestion in protein lysates (Figure 6I), confirming the direct binding of okanin with SIRT3. We hypothesized that okanin downregulates HaCaT cells exposed to UVA injury through regulating the SIRT3 pathway.

### 3.11. Okanin Increased Key Protein Levels in the SIRT3/FOXO3a Pathway

**Figure 7:**
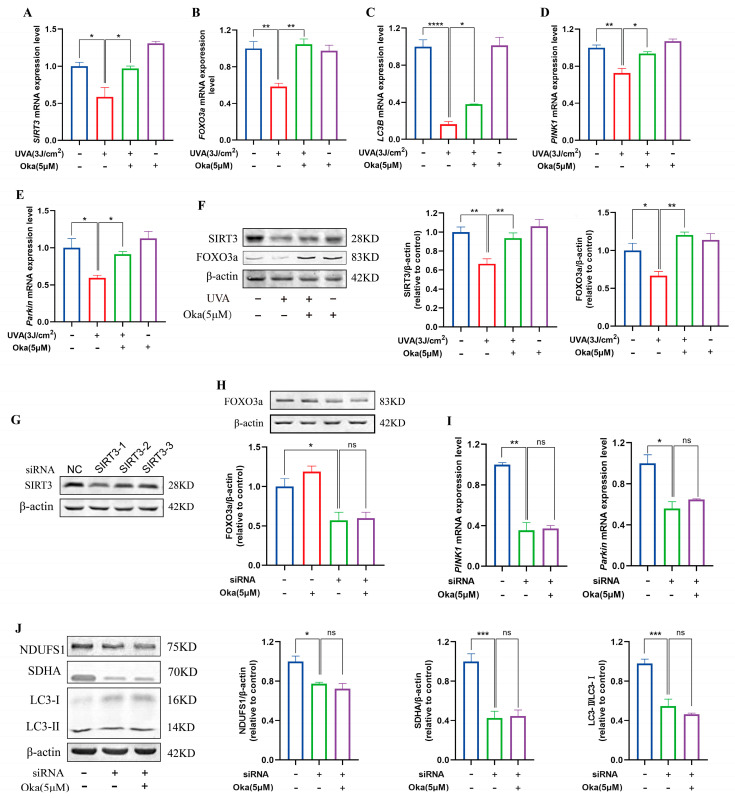
The effect of okanin on the SIRT3/FOXO3a signaling pathway. (**A**–**E**) The mRNA expression levels of *SIRT3*, *FOXO3a*, *LC3B*, *PINK1*, and *Parkin* were quantified by RT-PCR (n = 3). (**F**) The expression levels of SIRT3 and FOXO3a proteins. (**G**) siRNA inhibited the expression of the SIRT3 protein in HaCaT cells. The expression of the FOXO3a protein (**H**), and the expression of *PINK1* and *Parkin* mRNA (**I**) after SIRT3 knockdown using siRNA. (**J**) The expression of NDUFS1, SDHA and LC3-II/I proteins after SIRT3 inhibition. Values are presented as the mean ± SEM. * *p* < 0.05, ** *p* < 0.01, *** *p* < 0.001, **** *p* < 0.0001.

As displayed in Figure 7A, okanin pretreatment elevated *SIRT3* mRNA expression in UVA-exposed HaCaT cells. Subsequently, an analysis of SIRT3 pathway target genes revealed that okanin upregulated *FOXO3a, LC3B, PINK1,* and *Parkin* mRNA expression (Figure 7B–E). To assess the dependency of okanin’s protection on SIRT3/FOXO3a induction, we examined protein expression levels. The results confirmed that decreased SIRT3 and FOXO3a expression with UVA exposure could be reversed by okanin treatment (Figure 7F).

To verify the function of SIRT3 in okanin-mediated protection, we established a SIRT3 knockdown HaCaT cell model using siRNA. The result of Figure 7G confirmed successful SIRT3 depletion. Meanwhile, the expression of the FOXO3a protein reduced in SIRT3 knockdown cells, which was not reversed by okanin treatment (Figure 7H), and okanin failed to rescue *PINK1* and *Parkin* mRNA downregulation (Figure 7I). The knockdown of SIRT3 impaired the expression of mitochondrial complex proteins NDUFS1 and SDNA, as well as autophagy protein LC3-II/I, and treatment with okanin did not reverse this reduction (Figure 7J).

### 3.12. Okanin Improved Both Visible and Histological Signs of Photoaging in Mouse Skin

**Figure 8:**
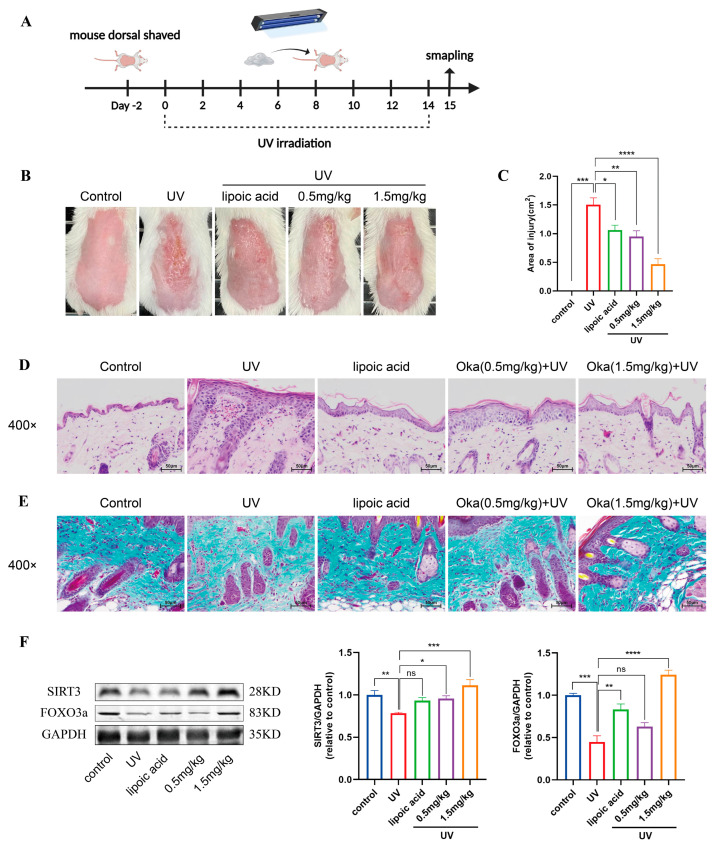
Okanin improves the macroscopic appearance of and histological changes in skin in photoaged mice. (**A**) A schematic diagram of UV irradiation mice model construction and drug administration. (**B**) Macroscopic changes in the dorsal skin of mice. (**C**) The area of skin damage on the back of mice (n = 8). (**D**) Representative sections stained with H&E (n = 3) (scale bar: 50 μm). (**E**) Masson’s trichrome staining detected a loss of dermal collagen fibers in the skin of mice (n = 3) (scale bar: 50 μm). (**F**) The expression levels of SIRT3 and FOXO3a proteins in the back skin of mice. * *p* < 0.05, ** *p* < 0.01, *** *p* < 0.001, **** *p* < 0.0001.

We developed a UV radiation mouse model to evaluate the anti-photodamage effects (Figure 8A). UV-irradiated mice exhibited characteristic photodamage including sunburn, dryness and desquamation. However, okanin-containing cream treatment ameliorated these cutaneous manifestations, reducing both injury severity and the affected area (Figure 8B,C).

Epidermal thickness has been shown to serve as a quantitative measure for the evaluation of skin inflammation and photoaging [28]. Next, we assessed UV-induced epidermal changes in mice using H&E staining. UV irradiation increased epidermal thickness, with concurrent dermal inflammatory infiltration and vascular dilation (Figure 8D). Collagen is essential for maintaining skin structure. Masson’s trichrome staining of mouse skin revealed sparse, disorganized dermal collogen (Figure 8E). However, okanin-containing cream mitigated UV-induced skin injury in mice, evidenced by a reduction in epidermal hyperplasia and dermal inflammation, as well as the restoration of collagen organization. The Western blot results showed reduced SIRT3 and FOXO3a protein expression in UV-irradiated mice skin, which okanin treatment restored (Figure 8F).

### 3.13. Okanin Prevents UVA-Induced DNA Injury and Cell Apoptosis in HaCaT Cells

**Figure 9:**
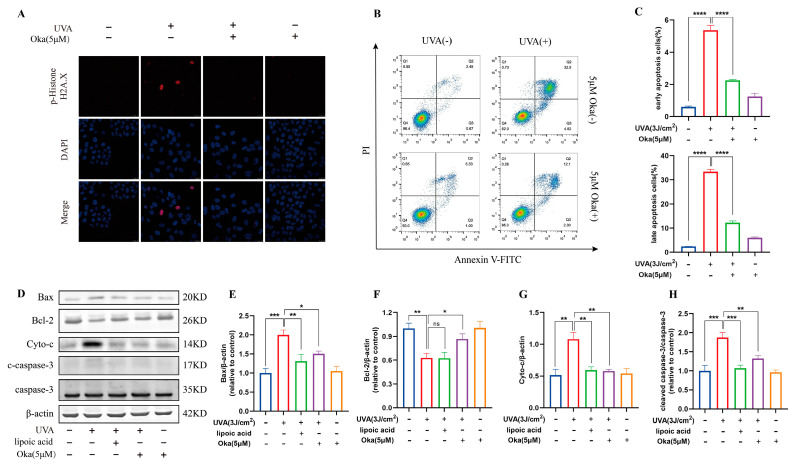
Okanin inhibits UVA-induced HaCaT cell apoptosis. (**A**) Phospho-Histone H2A.X was evaluated by immunofluorescence staining (Scale bar: 25 μm). (**B**,**C**) HaCaT cells were stained with Annexin V-FITC/PI and analyzed by using flow cytometry (n = 3). (**D**) Representative bands of Bax, Bcl-2, Cyto-c, caspase-3 and cleaved caspase-3 proteins. The corresponding quantification of Bax (**E**), Bcl-2 (**F**), Cyto-c (**G**) and cleaved caspase-3/caspase-3 (**H**). Values are presented as the mean ± SEM. * *p* < 0.05, ** *p* < 0.01, *** *p* < 0.001, **** *p* < 0.0001.

UV-induced DNA damage is a key contributor to photo-carcinogenesis and photoaging [29]. We found that HaCaT cells exposed to UVA irradiation induced significant DNA damage, demonstrated by improved phospho-Histone H2A.X positivity and staining intensity, however, okanin treatment significantly attenuated the increase of phospho-Histone H2A.X (Figure 9A).

Annexin V/PI staining indicated that okanin treatment reduced UVA-induced apoptosis in HaCaT cells (Figure 9B,C). Western blotting results further confirmed that okanin apparently downregulated Bax, Cyto-c and cleaved caspase-3 protein expression while elevating Bcl-2 expression (Figure 9D–H). These results indicate that okanin might protect against UVA-induced cell death by the mitochondrial apoptotic pathway.

## 4. Discussion

**Figure 10:**
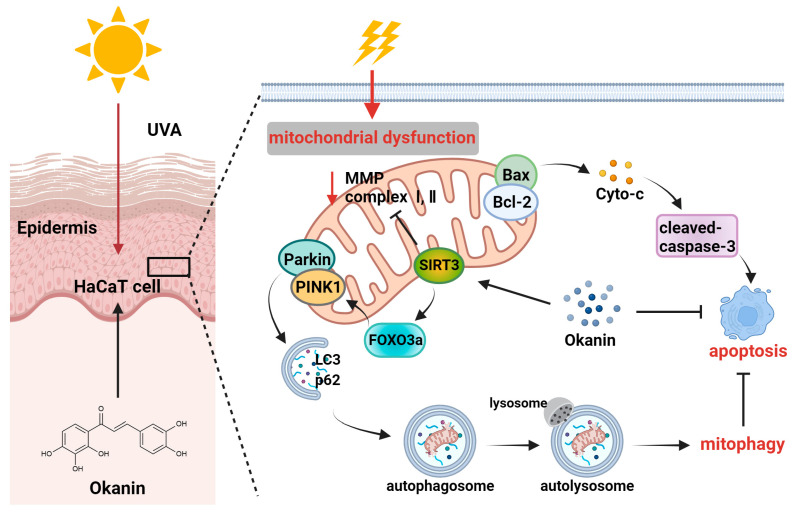
Mechanism of photoprotection by okanin against UVA-induced photoaging.

UV rays from sunlight are the most significant external factor in skin aging [30], and current lifestyles and climate changes have increased the vulnerability of the global population to UV radiation, which is also a major environmental pathogenesis of skin cancer [31]. With the increasing market demand for natural and multifunctional anti-skin aging products, research on the anti-aging activity of plant extracts has become a hot topic [32]. Okanin, a primary flavonoid in *Coreopsis tinctoria* Nutt., exhibits good antioxidant activity [33]. This study revealed the pharmacological mechanism of okanin in withstanding UVA damage using network pharmacology, along with in vivo and in vitro experiments. Figure 10 illustrates the molecular mechanism hypothesized in this study. We detected potential targets through the network pharmacology analysis of the TCMSP, ETCM, Herb, Swiss Target Prediction databases and Genecards. The complex interaction network of potential targets was constructed using STRING and Cytoscape, and the results indicated that *AKT1, SRC, SQSTM1 and BCL2L1* were potential therapeutic targets for UVA photoaging. Although *AKT* and *SRC* displayed high relevance (Figure 1E), the subsequent focus was directed toward targets with direct experimental and pathological evidence in UV-induced skin damage. The molecular docking analysis and DARTS assay determined SIRT3 as a target of okanin. This study elucidates the mechanism by which okanin mitigates UVA-induced photoaging, offering novel insights into its protective effects, partly highlighting its role in mitochondrial function, autophagy and apoptosis.

Environmental factors significantly contribute to skin aging, and UVA is the main source of ultraviolet radiation in human. A key mechanism of molecular responses in human skin initiated by ultraviolet radiation is the production of ROS, which also affects inherent aging processes [34]. However, excessive intracellular ROS accumulation poses significant harm, potentially contributing to multiple pathological conditions such as cancer, cardiovascular diseases, neurodegenerative diseases, and aging, including skin aging [35]. Enzymatic and nonenzymatic antioxidants make up the intracellular defense system to maintain ROS homeostasis [32,36]. When homeostasis is disrupted by excessive ROS, a pathological state termed “oxidative stress” is created in the cells, which damages intracellular macromolecules such as DNA, proteins, and lipids, etc. [37]. Apoptosis, abnormal immune responses and inflammatory responses are induced by these cellular and molecular alterations, etc. Studies have demonstrated UVA-induced ROS generation in human skin cells through both the direct detection of free radicals and indirect measurements including enzyme inactivation, DNA oxidative damage and GSH oxidation [36]. LDH release from UVA-irradiated HaCaT cells demonstrates membrane integrity loss and subsequent cell death [36]. In the present study, we found that after UVA irradiation, HaCaT cells showed decreased viability, increased LDH leakage and ROS production, whereas okanin decreased ROS production and increased cell viability. In addition, UVA-irradiated cytotoxicity was proved by the negative correlation of LDH leakage. Compared to ascorbic acid and butylated hydroxytoluene, Ma et al. reported that okanin exhibited stronger DPPH scavenging activities, and showed comparable cellular antioxidant activities to quercetin [38]. In our study, we found that okanin treatment improved GSH production and increased the T-AOC of the cells. Thus, the findings showed the protection of okanin against UVA-induced oxidative damage in HaCaT cells.

Mitochondrial dysfunction induced by UVR significantly contributes to skin photoaging [32]. Mitochondria, as primary producers of ROS, are especially vulnerable to oxidative stress, rendering mitochondrial function important in cell apoptosis and other age-related illnesses [39,40]. In our study, we found that the morphology of mitochondria was also altered. TEM observed that mitochondria pyknosis and cristae decreased after UVA irradiation. However, the okanin treatment counteracted UVA-induced mitochondrial damage, with increased mitochondrial content and improved morphology. It is worth noting that ROS primarily target the mitochondrial inner membrane, particularly complexes Ⅰ and Ⅱ. In our results, we found that UVA irradiation reduced mitochondrial complex Ⅰ/Ⅱ activity; moreover, it also downregulated NDUFS1 and SDHA protein expression in HaCaT cells. Importantly, okanin treatment ameliorated this phenomenon. The results provided further evidence of the protective effect of okanin in mitigating UVA-induced mitochondrial dysfunction.

Many studies have recently indicated that mitophagy plays an important role in cell senescence [14]. The PINK1/Parkin pathway is considered to be the most extensively studied mechanism of mitophagy [14]. Mitochondrial damage decreases membrane potential, triggering PINK1 accumulation in the outer mitochondrial membrane, thereby recruiting and activating Parkin through ubiquitin phosphorylation [11,41,42]. Subsequently, the Parkin-mediated ubiquitination of the outer mitochondrial membrane (OMM) protein enables an LC3-marked phagophore to engulf impaired mitochondria, resulting in mitophagosome formation [20,43]. In this process of mitophagy, cytosolic LC3Ⅰ undergoes conversion to form the membrane-bound LC3Ⅱ, which subsequently interacts with the autophagy adaptor p62/SQSTM1 for selective autophagic degradation [44,45]. As demonstrated by transmission electron microscopy, UVA radiation increased autophagosome formation in HaCaT cells, while okanin treatment significantly decreased autophagosomes. Moreover, in our research, the use of okanin increased the expression of LC3-Ⅱ/I, PINK1 and Parkin and decreased p62 protein expression. This suggested that okanin facilitated autophagy flow and promoted the further degradation of damaged mitochondria to delay aging skin.

SIRT3, an NAD+-dependent deacetylase, has been reported to regulate mitochondrial function and autophagy, making it a promising therapeutic target for skin aging [46]. As a nucleus transcription factor downstream of SIRT3, FOXO3 is activated via SIRT3-mediated deacetylation and then facilitates the transcription of various mitophagy genes, including PINK1. Therefore, SIRT3 could regulate mitophagy by mediating the FOXO3a/PINK1/Parkin signaling pathway [20,47,48]. SIRT3 deficiency induced ROS accumulation through cell apoptosis and anomalous autophagy, thereby deteriorating skin aging [46]. The results of molecular docking indicated the interaction of okanin with SIRT3 via pi–pi and hydrogen bonds. In addition, the SPR assay data showed an affinity of okanin for SIRT3 protein. Moreover, the DARTS assay, a universal target identification method, revealed that okanin directly binds SIRT3 by significantly enhancing its stability, resisting pronase digestion. Then, we illustrated that SIRT3 knockdown via siRNA reduced the expression of the FOXO3a protein. Furthermore, SIRT3 knockdown downregulated *PINK1* and *Parkin* mRNA expression, as well as LC3-Ⅱ/I, NDUFS1 and SDHA protein expression, which was an effect not reversed by okanin treatment. This indicated that the knockdown of SIRT3 could affect mitochondrial function and autophagy, and the protective effects of okanin was partially SIRT3-dependent. In accordance with the in vitro results, okanin elevated SIRT3 and FOXO3a levels in mouse skin, reduced UV damage through reduced wrinkles, improved skin hydration, normalized epidermal thickening, and enhanced collagen density.

In addition, it has been reported that cellular apoptosis is related to the regulation of Parkin-mediated mitophagy [49]. Mitochondrial dysfunction contributes significantly to pro-apoptotic protein release, including cytochrome C, which activates caspase proteins and induces apoptosis [50]. Many studies have found that UVA-induced cell apoptosis is related to the mitochondria-dependent (intrinsic) apoptotic pathway. In addition, UVA upregulates caspase-3 and caspase-9 expression, leading to relevant DNA damage [51]. This apoptosis process is mediated by Bcl-2 family proteins, particularly through the proapoptotic Bax and antiapoptotic Bcl-2/Bcl-xL [52]. In our findings, okanin exhibited protective effects against UVA-induced damage in HaCaT cells by alleviating cytochrome C release, enhancing Bcl-2 expression, and suppressing Bax and caspase-3 levels. The results of flow cytometry also demonstrated that okanin could improve UVA-induced apoptosis. The immunofluorescence analysis of HaCaT cells using Phospho-Histone H2A.X showed an increase in positive cells after UVA irradiation but a decrease in positive cells after okanin treatment.

Emerging evidence indicates that UV irradiation activates AKT in skin cells, leading to cell cycle arrest, the promotion of apoptosis, and the suppression of autophagy [53]. Sun et al. demonstrated that ARP (wild ginseng adventitious root protein mixture) significantly attenuated UVA-induced apoptosis, cell cycle arrest, and DNA fragmentation by enhancing AKT phosphorylation, concomitantly reversing UVA damage through Bax downregulation and Bcl-2 upregulation [54]. Furthermore, Src-family protein tyrosine kinases (SFKs) and the aryl hydrocarbon receptor (AhR) play pivotal regulatory roles in maintaining cellular homeostasis. Acute and chronic UV exposure in rodent skin induces aberrant Src phosphorylation, resulting in skin inflammation, photoaging, and skin cancer [55]. The PPI analysis revealed the high relevance of *SRC* and *AKT* to skin photoaging, suggesting the potential involvement of these targets in okanin resistance to UVA damage. However, our current investigation prioritizes targets with direct experimental and pathological validation in UV-induced skin damage. Thus, whether and how okanin affects AKT and SRC signaling to exert photoprotective effects warrants further mechanistic exploration.

## 5. Conclusions

In summary, in this study, we illustrated an important mechanism whereby okanin protected UVA-induced HaCaT cells from apoptosis and mitochondrial dysfunction, and this function was relevant to the clearance of the impaired mitochondria through partially regulating SIRT3/FOXO3a/PINK1/Parkin-mediated mitophagy (Figure 10). However, this study has several limitations that warrant consideration. When we explored the protective effect of okanin on mice exposed to UV radiation, our observations were primarily confined to the intuitive changes in the skin, as well as the histological level, and failed to further explore its underlying mechanism. In conclusion, the results indicated the potential of okanin as an active ingredient in skincare formulations, including skin creams, promoting the repair of skin under ultraviolet radiation.

## Figures and Tables

**Table 1:** si-SIRT3 sequences.

Genes	Forward	Reverse
*si-SIRT3-1*	5′-GGUGGAGAAGGUCCAUAUATT-3′	5′-AUAUGGACCUUCUUCCACCTT-3′
*si-SIRT3-2*	5′-GCAGAACAUCGAUGGGCUUTT-3′	5′-AAGCCCAUCGAUGUUCUGCTT-3′
*si-SIRT3-3*	5′-GGAAAGCCUAGUGGAGCUUTT-3′	5′-AAGCUCCACUAGGCUUUCCTT-3′

**Table 2:** Primer sequences.

Genes	Forward	Reverse
*SIRT3*	5′-TGCAGAAGTAGCAGTTCAGTG-3′	5′-GCTTCCTCTAGTGACACTGTTAG-3′
*FOXO3a*	5′-CGGACAAACGGCTCACTCT-3′	5′-GGACCCGCATGAATCGACTAT-3′
*LC3B*	5′-ATCACAGTTGGCACAAACGC-3′	5′-TACCCCTGCAAGAGTGAGGA-3′
*PINK1*	5′-CATGCCTACATTGCCCCAGA-3′	5′-GAACCTGCCGAGATGTTCCA-3′
*Parkin*	5′-TGGAGGGAAGAGAGGGGATG-3′	5′-GAATGCTGATGTGGCTGCTG-3′
*β-actin*	5′-AGAGCTACGAGCTGCCTGAC-3′	5′-AGCACTGTGTTGGCGTACAG-3′

**Table 3:** Cream components.

Component	
stearic acid	1.8 g
white petrolatum	2.0 g
liquid paraffin	1.3 mL
Tween-80	0.3 mL
glycerol	0.1 mL
distilled water	5 mL
okanin	0.002 g/0.006 g

## Data Availability

All data related to this research are presented in the manuscript.

## Supplementary Materials

The following supporting information can be downloaded at https://www.mdpi.com/article/10.3390/antiox14091040/s1, supplementary methods.

## Abbreviations

The following abbreviations are used in this manuscript:

GSH	glutathione
LDH	lactic dehydrogenase
MTT	methyl thiazolyl tetrazolium
ROS	reactive oxygen species
SPR	surface plasmon resonance
DARTS	Drug affinity responsive target stability
