# Supplementary material for: Okanin Attenuates Mitochondrial Dysfunction and Apoptosis in UVA-Induced HaCaT Cells by Mitophagy Through SIRT3 Pathway
Source: Antioxidants (Basel). 2025 Aug 23;14(9):1040. doi: 10.3390/antiox14091040 (PMC12466513; doi:10.3390/antiox14091040)
Supplement: Supplementary file 1 [file antioxidants-14-01040-s001.zip › antioxidants-3742845-supplementary.pdf]

Supporting Information

Okanin attenuates mitochondrial dysfunction and apoptosis in UVA-induced HaCaT cells by mitophagy through SIRT3 pathway

Fang Lu[1], Jiangming Zhong[2], Qi Zhou[2], Yiwei Yu[1], Mengdi Liang[1], Ying Yuan[1], Aowei Xie[1], Jin Cheng[1], Peng Shu[2,3,4,*], Jiejie Hao[1,*]

[1] Key Laboratory of Marine Drugs, Ministry of Education, School of Medicine and Pharmacy, Ocean University of China, Qingdao, 266003, China
[2] HBN Research Institute and Biological Laboratory, Shenzhen Hujia Technology Co., Ltd., Shenzhen, Guangdong, 518000, China
[3] State Key Laboratory Basis of Xinjiang Indigenous Medicinal Plants Resource Utilization, Xinjiang Technical Institute of Physics and Chemistry, Chinese Academy of Sciences, Urumqi, Xinjiang, 830011, China
[4] University of Chinese Academy of Sciences, Beijing, 100049, China

***Corresponding author, E-mail address:**
Peng Shu, shupeng@hbn.cn; Jiejie Hao, 2009haojie@ouc.edu.cn

Supplementary methods

Materials and reagents

DMEM, penicillin/streptomycin and FBS were purchased from Gibco (Grand Island, NY, USA). MTT assay, ROS assay, LDH, BCA protein assay kits were bought from Beyotime Biotechnology (Shanghai, China). RNAFit and siRNA were purchased from Han Hundsun Material Technology Co., LTD. Anti-β-actin (GB11001) and anti-GAPDH (GB15004) antibodies were bought from Sewell Biotechnology Co., Ltd. (Wuhan, China).

Main candidate targets of Coreopsis tinctoria Nutt. and drug-target-disease network construction

The drug-related databases, including Traditional Chinese Medicine Systems Pharmacology Database and Analysis Platform (TCMSP, https://old.tcmsp-e.com/), Encyclopedia of Traditional Chines Medicine (ETCM, https://www.tcmip.cn/ETCM/index.php), Herb Database (https://herb.ac.cn/), and Swiss Target Prediction (https://www.swisstargetprediction.ch/), were applied to gather the related targets of *Coreopsis tinctoria* Nutt. The targets obtained after excluding duplicates were imported into the STRING database and the Cytoscape 3.10.3 to expand the target range respectively, which could acquire the proteins corresponding to the intersection genes and the related proteins affecting their functions, and the first neighbors of selected nodes. The GeneCards (https://www.genecards.org/) was utilized to acquire the skin photoaging-related genes by skin aging, photoaging, inflammation, and mitochondrial autophagy as key words. Finally, the obtained genes between *Coreopsis tinctoria* Nutt. and skin photoaging using the Venn tools at https://jvenn.toulouse.inra.fr.

Functional enrichment analysis of intersection targets

For functional enrichment analysis of target genes, KEGG pathway, REACTOME pathway and WIKIPATHWAYS pathway enrichment were used, using a database for annotation, visualization, and integrated discovery (DAVID). The KEGG pathway, REACTOME pathway and WIKIPATHWAYS pathway enrichment were performed using the DAVID platform.

Construction of protein-protein interaction network (PPI)

PPI analyses were performed using the STRING database (https://stringdb.org/) to visualize the network of Coreopsis tinctoria Nutt. and photoaging. The species was set to Homo sapiens, and the protein-protein interaction (PPI) network was obtained. The PPI network TSV file was imported into Cytoscape 3.10.3 software to visualize the interactions of related target genes. Additionally, Cytoscape 3.10.3 was used to construct the network of drug-active ingredients, key targets, and core pathways.

Construction of drug-active ingredients-targets-pathway network

The app tool in Cytoscape 3.10.3 was used to analyze the active compounds and target information of *Coreopsis tinctoria* Nutt., and composite network was constructed based

on the point-to-line-to-point connection function.

Molecular docking

All protein structure files of SIRT3 were downloaded from RCSB PDB (https://www.rcsb.org/). The protein structures were prepared by DockPrep module and minimized with the ff14SB/gaff force field in UCSF Chimera (http://www.cgl.ucsf.edu/chimera/). Finally, all the structures were converted to pdbqt format with all hydrogens kept by rdkit2pdbqt.py script (https://github.com/biocheming/watvina, accessed on 26 December 2023, Ximing Xu, QingDao, China). The docking box size of each protein is set by extending ligand coordinate 5 Å on each dimension. Docking studies of SIRT3 structures and okanin were performed using watvina (https://github.com/biocheming/watvina, accessed on 26 December 2023 Ximing Xu, QingDao, China), which was developed by our group. Based on the molecular docking engine of Autodock vina, watvina is optimized on scoring function and conformation searching algorithm. The scoring function of watvina consists of Van der Waals, hydrogen bonds, polar-polar repulsion or hydrophobic attraction. Unlike Autodock vina, watvina considers the contribution of all hydrogen atoms. Conformation searching adopts a simplified genetic algorithm-simulated annealing-BFGS combination strategy; in addition, a torsion penalty is calculated for conjugated rotable single bonds.

SPR research

The SPR experiments were carried out using a Biacore T200 instrument (Biacore; GE Healthcare) at 25 °C. The CM5 chip surface was activated after mixing 0.4 mol/L EDC and 0.1 mol/L NHS at a 1:1 ratio. Next, the chip surface was blocked with ethanolamine (pH 8.5). The analytes and different concentrations of okanin diluted using PBSP buffer were injected and passed over the immobilized SIRT3 sensor surface. The flow rate was 30 μL/min, the binding time was 90 s, and the dissociation time was 90 s. The experimental data were analyzed using Biacore T200 evaluation software.

Drug affinity responsive target stability (DARTS) assay

HaCaT cells were seeded in 10-cm dishes, and protein was extracted with lysates at 80% confluency. After centrifugation (13,000 rpm, 10 min, 4°C), 10× TNC buffer [500mM Tris·HCl (pH 8.0), 500mM NaCl, 100 mM $CaCl_2$] was added to the lysates. Protein lysates were incubated with vehicle control (DMSO) or okanin at 4 °C for 12 h. For proteolysis, each cellular lysate sample was subjected to treatment with pronase (1:100 ratios of pronase vs. cell lysate proteins) at room temperature (RT) for 2 min. Digestion was stopped by adding 5× loading buffer, then the mixtures were denatured by boiling. The samples were subjected to Western blot analysis.

Immunofluorescence

For immunofluorescence labeling, HaCaT cells were seeded in confocal glass bottom dishes. After the corresponding treatment, the cells were washed once with PBS and fixed with 4% paraformaldehyde for 15 min. Subsequently, they were washed 3

times with PBS (5 min per wash) and permeabilized with 0.1% TritonX-100 in PBS for 5 min. After washing thrice, the cells were blocked with blocking buffer for 1 h at room temperature, and incubated with primary antibodies in a moist chamber at 4 °C overnight. Cells were washed 3 times with PBS and incubated with FITC-labeled secondary antibody for 1 h at 37 °C. After washing thrice with PBS, DAPI staining solution was added to stain the nuclei, and the cells were incubated at 37 °C in the dark for 15 min. After washing three times with PBS, the cells were observed under a laser confocal microscope.

Histological analysis

Dorsal skin samples (approximately 0.5×0.5 cm) were obtained by quick stripping on day 15, dehydrated in ethanol, embedded in paraffin, and sectioned. The sections were stained with H&E and Masson's trichrome stain. The histopathologic changes were examined under a light microscope.
